# Near-peer mentorship for undergraduate training in Ugandan medical schools: views of undergraduate students

**DOI:** 10.11604/pamj.2016.23.200.7691

**Published:** 2016-04-15

**Authors:** Godfrey Zari Rukundo, Aluonzi Burani, Jannat Kasozi, Claude Kirimuhuzya, Charles Odongo, Catherine Mwesigwa, Wycliff Byona, Sarah Kiguli

**Affiliations:** 1 Department of Psychiatry, Mbarara University of Science and Technology, Uganda; 2 Department of Nursing, Mbarara University of Science and Technology, Uganda; 3 Directorate of Academic Affairs, Kampala International University, Western Campus, Uganda; 4 Department of Pharmacology, Kampala International University, Western Campus, Uganda; 5 Department of Pharmacology, Gulu University, Uganda; 6 School of Dentistry, Makerere College of Health Sciences, Uganda; 7 Department of Paediatrics, Makerere College of Health Sciences, Uganda; 8 Research Consults, Kampala, Uganda

**Keywords:** Undergraduate medical education, near peer mentorship, Ugandan medical education

## Abstract

**Introduction:**

Masters Students are major stakeholders in undergraduate medical education but their contribution has not been documented in Uganda. The aim of the study was to explore and document views and experiences of undergraduate students regarding the role of masters students as educators in four Ugandan medical schools.

**Methods:**

This was a cross-sectional descriptive study using qualitative data collection methods. Eight Focus Group Discussions were conducted among eighty one selected preclinical and clinical students in the consortium of four Ugandan medical schools: Mbarara University of Science and Technology, Makerere College of Health Sciences, Gulu University and Kampala International University, Western Campus. Data analysis was done using thematic analysis. Participants’ privacy and confidentiality were respected and participant identifiers were not included in data analysis.

**Results:**

Undergraduate students from all the medical schools viewed the involvement of master's students as very important. Frequent contact between masters and undergraduate students was reported as an important factor in undergraduate students’ motivation and learning. Despite the useful contribution, master’ students face numerous challenges like heavy workload and conflicting priorities.

**Conclusion:**

According to undergraduate students in Ugandan medical schools, involvement of master's students in the teaching and learning of undergraduate students is both useful and challenging to masters and undergraduate students. Masters students provide peer mentorship to the undergraduate students. The senior educators are still needed to do their work and also to support the master's students in their teaching role.

## Introduction

Masters students often get involved in the teaching and learning of undergraduate students in medical schools worldwide [1–3]. Teaching can be a valuable experience for masters students and the undergraduate students [4]. Those teaching in clinical disciplines form the back-borne to service provision and supervision of undergraduate students. In addition, masters students could influence the undergraduates in choosing a career since they may act as role models. It can also contribute to intellectual development [4, 5] and to the individual′s grasp of the subject. The involvement can also provide an opportunity for career development for the masters students with a plan to remain involved in medical education. Participating in undergraduate teaching can substantially enhance the master's student's life skill development, a sense of confidence and responsibility [6, 7]. However, the teaching of undergraduate students can also be challenging to the masters students in several ways: too much teaching could erode research time and limit the master's student′s capacity to complete his/her own thesis and prolong their periods of study. The benefits and challenges encountered in the process have also not been studied and documented in the developing world. Research in the developing world has not have considered medication education a priority research area.

In Uganda, mentorship is not yet fully institutionalized in medical schools. But, the mentors and mentees find themselves in a situation where learning may take place. Many of the mentors and mentees may not know their roles and responsibilities [8]. Many times, the masters students are involved in the training and mentorship of undergraduate students. This is not a common experience in the developed world but may be useful in the developing world. Considering the use of mentors with higher qualifications may not be realistic in an environment with very low staffing levels and heavy workloads. This study explored and documented the views and experiences of undergraduate students regarding the role of masters students as educators in four Ugandan medical schools.

## Methods

**Study design**: this was cross-sectional descriptive study using qualitative data collection methods. The study was conducted at four Ugandan medical schools in the MESAU (Medical Education for equitable Services to All Ugandans) consortium comprising Mbarara University of Science and Technology (MUST), Makerere College of Health Sciences (MakCHS), Gulu University and Kampala International University, Western Campus (KIU). The MESAU consortium was started with the aim of improving the quality of medical education and increasing the number of health professionals in Uganda.

**Study participants**: the participants were eighty one (n = 81) preclinical and clinical students pursuing various undergraduate programmes offered in the medical schools. The programmes included MBChB, Nursing, Medical Laboratory Sciences (MLS) and Dentistry.

**Data collection**: eight Focus Group Discussions (FGDs) were conducted among eighty one (n = 81) preclinical and clinical students in four Ugandan medical schools in the MESAU consortium: Mbarara University of Science and Technology, Makerere College of Health Sciences, Gulu University and Kampala International University, Western Campus. Two FGDs were conducted at each of the four medical schools; one with preclinical and the other with clinical students. Student leaders were contacted and asked to mobilize ten students from various programmes and years of study that were willing to participate in the study. Four of the eight FGDs were conducted by the first author and the seventh author. Of the remaining FGDs, two were conducted by the first author and the forth author. The other two were conducted by the fourth, fifth and the seventh authors. For all the FGDs there were research assistants supporting the process and taking notes. Each of the FGDs had ten participants except one which had eleven. The participants were both male and female. In addition, Key Informant Interviews (KIIs) and the In-Depth Interviews (IDIs) were also conducted among senior educators and masters students at the same medical schools respectively. The views and experiences of masters students and the senior educators are not the focus of this paper; they are described in another paper. This study was approved by the Institutional Research and Ethics Committee of Mbarara University of Science and Technology (MUST-REC). Participants′ privacy and confidentiality were respected and participant identifiers were not included in data entry and analysis.

**Data management and analysis**: during interviews and focus group discussions, documentation of data was crucial, information was always captured by writing down notes, capturing voices with the assistance of recorders and later this data was transcribed in a verbatim format. The quality of transcription was monitored against the actual recorded voices to maintain and ensure consistency. Data analysis was done manually by identifying emergent themes which were later coded and organized into concepts which were later developed in to tentative explanations but this was attained after reading through the data several times line by line to get a holistic picture out of it. This further gave the researchers opportunity to familiarize themselves with the data. Diagramming the relationship among concepts was done to demonstrate how one concept influences another so as to generate generalization, trends and conclusions.

## Results

### Socio-demographics and programme representation

The undergraduate students that participated in this study were from the four medical schools in the MESAU consortium. There were 30 female students and 51 male students. There were from different programmes including MBChB, Nursing, Medical laboratory science, dentistry. More than 70% of the participants were medical students. The medical students seem have more contact with the masters students compared to students in other programmes. In addition, the medical students are the majority in the medical schools. All the medical schools have equal numbers of students (20 per institution) participating in the study. Participants were selected from preclinical and clinical years to reflect adequate representation of the medical school.

### Near peer mentorship between masters and undergraduate students

Undergraduate students from all the medical schools viewed the involvement of masters students as helpful and beneficial to them but also to the masters students. They viewed masters students as mentors who are close to their level in terms of age and professional complexity. In sharing their views and description of their experiences, they often compared them with the senior educators. They also gave personal experiences.

“essentially when a student is teaching a student, they would always find it easier to teach the same people because they are almost going through the same similar systems and I would say, they wouldn't seem aloof like some of these senior old lecturers, at times “not to disrespect any of them but at times. They seem like… they are in their own world… they left this whole confusion of studying of ours” *(Preclinical student, MakCHS). The undergraduate students also reported that the experiences may not cut across all programme and all masters students. They said the experiences were different with different masters students.*

“During my journey of education… …, “I have learnt a lot, there are some people who are teachers naturally and there are some people no matter how much data they have, they are really bad”. *(Clinical student, Gulu)*.

“To me, I think it is good but depends on the personality of that particular masters because there are some who I have met who are good and there are some who are not good” *(Preclinical student, MakCHS)*.

“Another thing is that some of them are really approachable because we are all students so we can know when to approach them. There are times we fear to approach lecturers… at times, the lecturer might be a “mzee” (referring to an elderly person) and then you wonder what you want to get from this mzee… Laughters… from other participants… so it becomes tricky but if it is a fellow student though being ahead of me, we can discuss freely, may be outside, so they can model me up and put me at the right track” *(Preclinical student, Gulu)*.

Many of the undergraduate students considered the masters students to be endowed with fresh knowledge that can be beneficial to the undergraduate students.

“My experience with them (masters students) is that they seem to be fresh so they try to put a lot of attention in transforming what they read into practice; for example in the years we were doing anatomy. You would see that they were putting a lot of emphasis for you to see what we read in the books so I feel that actually the committed ones are really doing a good job of teaching their students”.

In the current study, there were differing views concerning the comparison of seniors and masters students in teaching. Some of the students preferred the senior educators where as others preferred the masters students.

“I think the information given by the seniors is well packaged, by the time one is appointed a lecturer, you must be good. Usually you find themasterss could be having the information but do not give it to you appropriately. So, much as these masterss give us information; I would rank seniors as much better”. *(Clinical student, MUST)*.

I have this feeling that when a senior is coming to lecture, you will be so much motivated for example if it is medical ward and professor is coming, there is no way you can fail to go there, so there is always that feeling of, “I am really going to gain a lot”. They teach you what is appropriate for you to learn *(Clinical student, MUST)*.

“Senior lecturers and professors have more content so when they are explaining the concept we try to grasp them for example for us in bio chemistry, if professor Gertrude lecturers, you understand everything very well in that even if you go for exams, you can easily remember whatever she taught but when it comes to masters students, they just come and rush through the lecture and make you more confused than when you came in” *(Pre-clinical student, MUST)*.

### Masters students boosting human resource in the medical schools

It was general consensus that there were few senior staff in all the medical schools in Uganda and that masters students made a big contribution to boost human resource. They were alos considered to bridge the gap between the senior educators and the undergraduate students.

“Well I think these masters students are the only bridge between us and the senior lecturers, they can talk to them, they can also talk to us, and they are always in the middle” *(Preclinical student, MakCHS)*.

“Another thing the senior lecturers have is that they are always very busy, they have workshops to attend to, research, conferences so you have to wait for them to come but for the masters, they are always around” *(Preclinical student, MakCHS)*.

“Okay for me I agree for them to teach because they are always available unlike the senior lecturers who rarely attend lectures, but for them they are always available, they are friendly, you can learn from them” *(Clinical student, Gulu)*.

“one can even spend some time without seeing their lecturers like for us, we are in third year and we are in the gynecology ward but have not seen them at all so I do believe that if these masters students are there, they can help to pass on some information” such as in theory because from my experience, I was interacting with a student who was doing surgery from Makerere, he was ever with me at least giving me that support, in your free time, he would help, take you to the theatre, helps you to organize a patient. The challenge is that the senior lecturers do not have time for us, that is what I can say, they are always busy with their things. Then as per the issue of time, I think these masters students do it out of their will, they are not facilitated, it is a personal initiative”.

### Masters students benefit from their teaching role

According to undergraduate students, the masters students benefit from their teaching role; it helps them to better their skills as future educators. In addition, the masters students get the opportunity to grasp a topic they are teaching to undergraduate students (Table 1).

**Table 1:** Codes, categories and themes on undergraduate students’ experiences of the involvement of masters students in undergraduate training in Ugandan medical schools

Codes	Categories	Themes
**Graduate students appreciated**		
Age gap and level gap are narrow between graduate students and undergraduate students, and they identify with one another. They also provide Research mentorship	Narrow gap facilitates learning	Near peer mentorship between graduate and undergraduate students
It is easier for Graduate students to share even non-academic opportunities		
Graduate students are up-to-date with information/knowledge and they simplify concepts for example providing mnemonics to aid learning.	Graduate students may be more understood compared to senior educators	
Graduate students give more comprehensive content		
Graduate students have more time for undergraduate students than seniors	Graduate students considered very useful in imparting clinical skills	
Graduate students have more hands-on/practical experience		
**Graduate students Bridging the gap**		
Graduate students are the bridge between Seniors and undergraduate students	Graduate students Boost human resource	Graduate students Boost human resource
Graduate students supervise and guide/provide mentorship	Graduate students are available to undergraduates	
Graduate students boost the limited teaching staff and are always available to teach		
**Benefits to graduate students**		
Graduate students grasp facts for their benefit as they teach undergraduate students	Graduate students benefit from the teaching in a way of revision	Graduate students benefit from their teaching role
Graduate students also develop confidence as they teach undergraduate students	Involvement of graduate students in assessments/exams is helpful to them as they prepare for their own exams	
Teaching is a way of catching up for graduate students and gives them opportunity to explore more

“I think by teaching us, they gain more experience in teaching and then secondly, they also gain some knowledge from us which is good” *(Clinical student, MakCHS)*.

I think in learning and teaching, these postgraduate students benefit from us and we also benefit from them, if you teach others, you learn *(Clinical student, MUST)*.

“It depends, I think it would be good for the masters students to teach us, you see when you verbalize something, you cannot forget it, so I think they benefit a lot” *(Clinical student, MUST)*.

### Masters students face Challenges in their role as educators

Despite the useful contribution they make towards undergraduate teaching and learning, masters students face numerous challenges (Table 2).

**Table 2:** Codes, categories and themes on challenges faced during masters students involvement in undergraduate training in Ugandan medical schools

Codes	Categories	Themes
Graduate students are usually not trained as teachers and may not explain well what they teach. They may not know their limits and may give too much information	Lack of training in teaching methods is one of the challenges faced by graduate students while teaching undergraduate student	Lack of preparation for their role as educators
Limited confidence making graduate students insecure resulting in their becoming rude/harsh to undergraduate students	Lack of confidence and experience	Heavy workload with no motivation
Due to lack of experience, they may complicate otherwise easy- to- comprehend work.		
After being overwhelmed/stressed by excessive workload, they become rude/harsh to undergraduate student and having negative attitudes	Heavy Workload for graduate students	
Unsynchronized time-table for graduate student and undergraduate student leading to unavoidable clashes and missed teaching or exam supervision	No motivation/pay for Graduate students	
Some departments don't have organized teaching schedules for graduate students, Graduate students may be unable to accomplish their studies as they devote enough time to teaching undergraduate students	Graduate students having limited time; unable to accomplish their studies and devote enough time to teaching undergraduate students	
Some graduate students lack commitment and passion for teaching; only doing teaching as an obligation

“Some of these masters students just down load information from the internet, something you can easily notice with your eyes. So at the end of the day, important topics are made hard and yet they are simple”. Actually for me I like the Psychiatric ward, all the people who are handling us are seniors, there are no masters students teaching us, they know what they are doing. I used to read ahead but you would find that a senior lecturer would just break down the topic very well for you and “i would Say… YES. This is it”, so I will go on in support of senior lecturers teaching us” *(Clinical student, MUST)*.

### What could be done differently to Support Masters students in their role as educators?

In order to enhance the role of masters students as educators, some things have to improve or have to be done differently (Table 3).

**Table 3:** Codes, categories and themes on undergraduate students’ recommendations concerning the involvement of masters students in undergraduate training in Ugandan medical schools

Codes	Categories	Themes
Graduate students should have formal preparation before asking them to teach undergraduates	Need to prepare graduate students for the teaching role	Need to do things differently in order to support graduate students
Graduate students need support from seniors in teaching.	Need for support supervision for graduate students from seniors	
Seniors should not leave teaching for graduate students.		
Graduate students doing teaching as an obligation but lacking desire to teach/interest due to no motivation	Need for payments/motivation for graduate students when they teach	
Need to synchronize the timetables for graduate students and undergraduate students	Timetabling for graduate and undergraduate students should be integrated

However, I don't know whether this is because of lack of motivation, there was a masters student who almost made students fail exams because he was not teaching us, he said “After all, I am not paid” *(Clinical MUST)*

“My suggestion is that supposing it was made part of the curriculum that these people score some marks, because as a Doctor, the Hippocratic Oath requires that you teach, so when you refuse to teach undergraduates, I wonder how they can benefit. So if they could earn a mark from the way they interact with the students and the juniors, then may be these masters students would be more interested to go and teach. As a medical person, it is a requirement to go and teach. So my view is that they attach some kind of reward or requirement while they are teaching undergraduates” *(MUST Clinical)*.

“Many of these masters students are self-sponsored while others are sponsored by organizations and they seem motivated but when you meet somebody who is self-sponsored, they tend not to be free with information because they also have issues that make them think of better ways of raising money, their attitude depends on the financial support that they receive” *(MUST clinical student)*.

“I think they should really be motivated so that they can be able to pay their tuition, I doubt if there is any government program sponsoring them” *(Clinical student, Gulu)*.

## Discussion

This study explored and documented the views and experiences of undergraduate students concerning the role of masters students as educators in four Ugandan medical schools. We found that masters students make a valuable contribution to the teaching and learning of undergraduate students in all the four medical schools. Their contribution is appreciated by the students who view them as better mentors who are close to them and more available for learning to take place. The study realized that masters students need support and training from the senior educators in order to be able to do a better job. Involvement in teaching benefits both masters and undergraduate students. Masters students boost the manpower in the four medical schools which are understaffed in majority of the departments. However, involvement in teaching/learning of undergraduate students is challenging to both the masters and undergraduate students [9].

The findings in this study compare with previous studies. For instance, Evans et al [10] reported that involving senior students teach junior students was beneficial to both the teachers and the students, The success of the Peer teaching and mentorship have been considered to be due to effective communication between the students and the peer mentors. This relationship has been associated with improved the learning and skills development. As the peer teachers/mentors support their fellow students, they also reinforce their own learning and develop essential teaching skills for future use [10]. The student-student mentorship provides an effective environment for developing deeper learning different subjects through teaching. The same principle seems to work between junior teachers mentoring each other [11–13].

Although the masters students are important in mentoring undergraduate students, they should not replace the seniors in the teaching responsibility. Seniors should be present to support the masters students and also correct any mistakes that masters students may make. Recognizing that masters students also have their own needs as students, there is need for an organized plan to mentor them and also prepare them for the teaching role. Masters students need appropriate feedback on performance by assessing the existing knowledge and competence. The masters students and the senior educators involved in the training and mentoring of undergraduate students need to be trained for the task [14]. There are special skills needed for successful training to take place [15]. Providing skills to masters students in preparation for their involvement in undergraduate training may improve their retention in the training institutions [16].

## Conclusion

The project described was supported by Award Number 1R24TW008886 (MESAU-MEPI Programmatic Award) from the Fogarty International Center. The authors also appreciate the study participants for their time and contributions and the research assistants for their hard work.
